# Case Report: Induction of labor and postpartum management for fetal cardiac rhabdomyoma: a genetic etiology-based analysis of three cases

**DOI:** 10.3389/fped.2026.1822341

**Published:** 2026-07-09

**Authors:** Xinying Liu, Hongwei Fu, Qinggui Ren, Hua Shu, Guanchen Wu, Huiming Xu, Xueqin Feng

**Affiliations:** 1 Department of Obstetrics, Affiliated Hospital of Jining Medical University, Jining, China; 2 College of Clinical Medicine for Obstetrics & Gynecology and Pediatrics, Fujian Medical University, Fuzhou, China; 3 Department of Mammary Gland Surgery, Affiliated Hospital of Jining Medical University, Jining, China; 4 Department of Clinical Medicine, Jining Medical University, Jining, China

**Keywords:** fetal rhabdomyoma, genetic stratification, induction of labor, perinatal management, tuberous sclerosis complex (TSC)

## Abstract

Fetal cardiac rhabdomyoma (CR) is a rare primary cardiac tumor often linked to tuberous sclerosis complex (TSC) caused by TSC1/TSC2 mutations, yet its genetic heterogeneity and individualized perinatal management remain incompletely defined. This case series reports three primigravid women with fetuses diagnosed with CR at different gestational ages, who received induced labor via various regimens. Genetic testing identified a paternally inherited TSC2 mutation in one case, a *de novo* TSC1 mutation in another, while genetic analysis was declined in the third. Distinct differences exist in recurrence risk, long-term neurological prognosis and corresponding clinical decision-making between *de novo* TSC1 variants and paternally inherited TSC2 mutations, which fully reflects prominent genetic heterogeneity among TSC-related fetal CR. This study demonstrates the clinical and genetic heterogeneity of fetal CR, and highlights that ultrasound evaluation, TSC1/TSC2 genetic testing, and multidisciplinary care are crucial for guiding induction timing, perinatal management, and genetic counseling to improve perinatal outcomes.

## 1 Introduction

**Figure 1:**
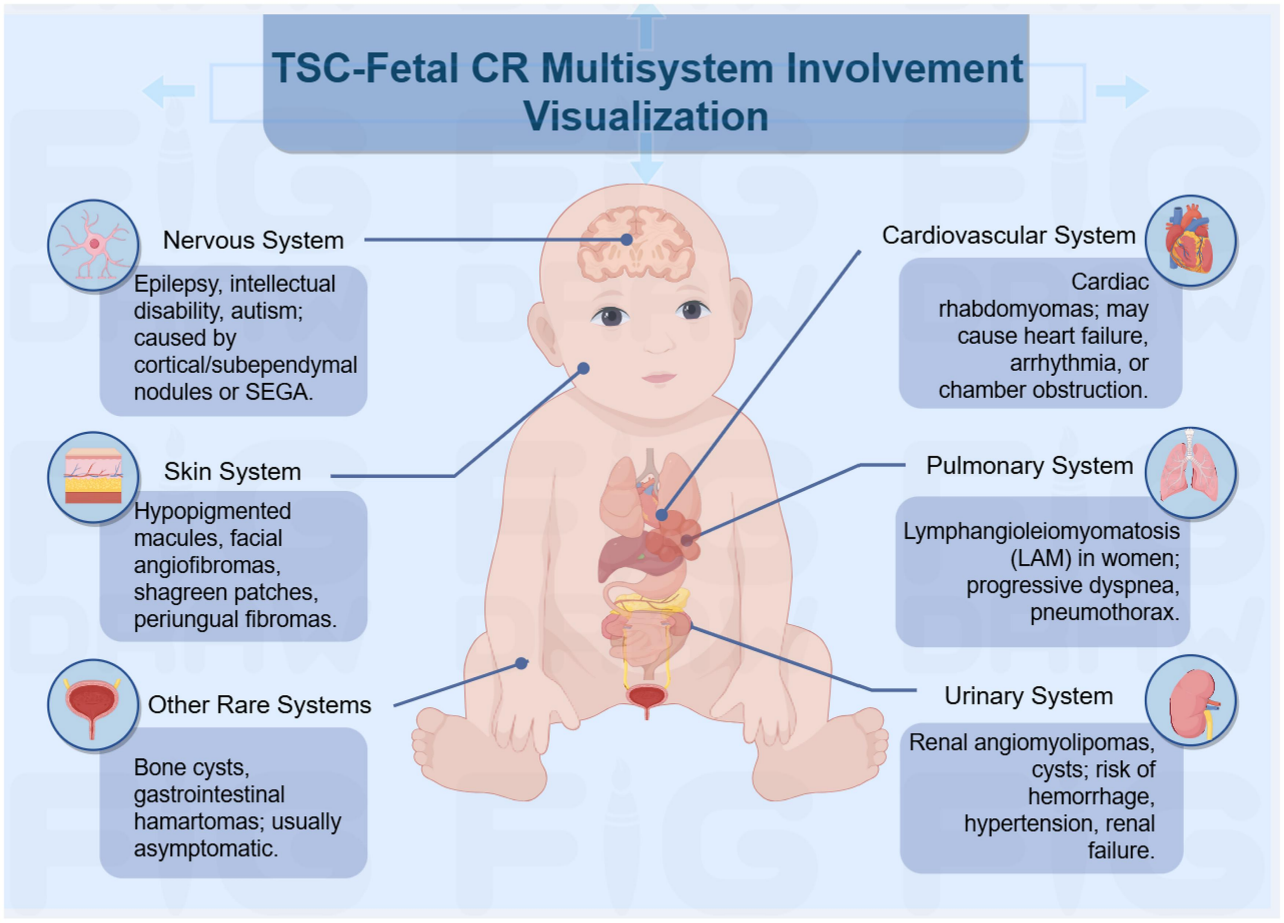
Tuberous sclerosis Complex (TSC)-associated fetal cardiac rhabdomyoma (CR): multisystem clinical manifestations. This schematic illustrates the diverse organ involvement in TSC, emphasizing systemic manifestations that may co-occur with fetal cardiac rhabdomyomas. Key systems include the nervous system (epilepsy, intellectual disability, subependymal nodules), skin (hypopigmented macules, angiofibromas), cardiovascular system (cardiac rhabdomyomas), pulmonary system (lymphangioleiomyomatosis in women), urinary system (renal angiomyolipomas), and other rare sites (bone cysts, gastrointestinal hamartomas).

Fetal cardiac rhabdomyoma (CR) is the most common primary cardiac tumor affecting fetuses and neonates, with a prevalence of approximately 0.1–0.2 per 1,000 live births (1). It is classified as a hamartoma rather than a true neoplasm, with a proposed origin from fetal cardiac myoblasts. Approximately 75% of CR cases are associated with tuberous sclerosis complex (TSC), an autosomal dominant genetic disorder caused by mutations in the TSC1 or TSC2 gene (2). TSC is characterized by multi-organ hamartomas involving the brain (often leading to epilepsy or intellectual disability), skin, kidneys, and heart (Figure 1). A subset of CR cases may occur sporadically and may also be association with structural chromosomal aberrations (3).

Prenatal transabdominal ultrasound constitutes the first-line diagnostic modality for fetal CR, which characteristically presents as well-circumscribed hyperechoic intracardiac nodules. Differential diagnosis requires rigorous exclusion of other space-occupying lesions such as cardiac fibroma and hemangioma. The distinction between TSC-related and non-TSC-related CR represents a pivotal component of clinical assessment, as this stratification directly determines prognostic evaluation and familial genetic counseling. Genetic testing for TSC1/TSC2 pathogenic variants is therefore strongly recommended in clinically suspicious cases (4).

Comprehensive perinatal management for CR-affected pregnancies requires integrated evaluation of multiple factors, including tumor size, number, location, cardiac functional impact and associated TSC status, as well as their cumulative effects on fetal and neonatal cardiovascular function. Decisions concerning pregnancy continuation, induction timing, delivery mode and postpartum care should accordingly be guided by a multidisciplinary team comprising obstetricians, cardiologists, clinical geneticists and neonatologists. This approach is fully concordant with the 2019 Fetal Tumor Management Guidelines (5). Given the inherent complexity of fetal CR, this entity remains a central focus in perinatal medicine and prenatal genetics. Timely diagnosis and individually tailored management are indispensable for optimizing maternal and neonatal outcomes.

## 2 Case report

### 2.1 Case 1

**Figure 2:**
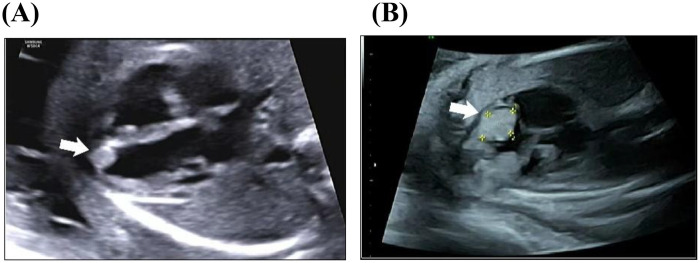
Prenatal ultrasound images of fetal cardiac rhabdomyomas (CR) in Two cases. **(A** Case 1: White arrow indicates multiple hyperechoic CR nodules in the fetal bilateral ventricles. **(B)** Case 2: White arrow indicates multiple hyperechoic CR nodules in the fetal bilateral ventricles, with the larger one (1.3 × 1.0 × 1.2 cm) in the right ventricle.

A 25-year-old G1P0 female was admitted for induction due to “detection of fetal cardiac hyperechoic nodules at 27 + 2 weeks of gestation”. She had regular 30-day menstrual cycles, with the last menstrual period confirmed via early pregnancy ultrasound. She did not attend regular prenatal visits during pregnancy. Maternal serum Down syndrome screening, nuchal translucency (NT) screening, and glucose tolerance testing (GTT) at an external hospital were all normal. S Transabdominal ultrasound at a local hospital first revealed a hyperechoic nodule in the fetal left ventricular apex. Our hospital's ultrasound confirmed the finding, suspecting CR. Prenatal diagnosis (amniocentesis) was recommended but declined by the patient (Figure 2A). At admission, she reported no abdominal pain or vaginal bleeding, with a total pregnancy weight gain of 7 kg. The pregnancy risk assessment was classified as orange (indicating moderate pregnancy risk).

#### 2.1.1 Past history

She had a 2-year history of polycystic ovary syndrome (PCOS), managed with oral contraceptives, and carried a balanced chromosomal karyotype of 46,XX,t(6;11)(p21.3;p11.2), which provides a typical clinical sample for analyzing the relationship between maternal chromosomal structural abnormalities and fetal CR etiology. She underwent hysteroscopy for endometrial polypectomy in 2022. Her husband was a TSC2 gene heterozygous carrier.

#### 2.1.2 Diagnostic/treatment process and follow-up

Multidisciplinary evaluation was performed by specialists in maternal-fetal medicine, fetal cardiology, clinical genetics, and neonatology. The family received extensive counseling regarding the high suspicion of TSC-associated CR, the dismal long-term neurological prognosis, and the elevated recurrence risk. Although the family had initially declined amniocentesis, they firmly requested termination of pregnancy (TOP) following comprehensive consultation. Following admission, induction was performed via amniocentesis with 100 mg rivanol (lactic acid ethacridine injection), followed by vaginal delivery of a stillborn male infant. The placenta was completely delivered, while the fetal membranes were basically retained. Postpartum, 10IU oxytocin (intravenous drip) and 100*μ*g carbetocin (intramuscular injection) were administered to promote uterine contraction. The family requested fetal skin tissue sampling for TSC1/TSC2 single-gene testing. Postpartum day 2 follow-up ultrasound revealed intrauterine residual tissue with a maximum diameter with 1.5 cm. The patient declined uterine curettage and received conservative treatment with oral Guizhi Fuling Capsules. One week later, repeated ultrasound showed that the intrauterine residue had reduced to 0.8 cm, with satisfactory uterine involution. Genetic testing identified a heterozygous c.2286dupG mutation in the fetal TSC2 gene, which was consistent with the paternal genotype, thereby establishing a definitive diagnosis of TSC-related CR.

### 2.2 Case 2

A 30-year-old G1P0 female was admitted for induction at 38 + 1 weeks of gestation due to “multiple hyperechoic nodules in the fetal heart”. She had irregular menstrual cycles (30–60 days), with gestational age confirmed via early pregnancy ultrasound. NT screening and non-invasive prenatal testing (NIPT) for common aneuploidies were normal. GTT was not performed due to patient refusal. Transabdominal ultrasounds at a local hospital and our institution both showed “multiple hyperechoic nodules in the fetal left and right ventricles, the larger lesion located in the right ventricle with size about 1.3cm × 1.0cm × 1.2 cm, clear boundary, heterogeneous internal echo (possible CR)”(Figure 2B). The routine prenatal systematic ultrasound detected no obvious intracranial space-occupying lesions or subependymal nodules suggestive of TSC. The patient and her family opted for pregnancy termination and refused genetic testing. At admission, fetal movements were normal, with a total pregnancy weight gain of 15 kg. The pregnancy risk assessment was classified as yellow (indicating low pregnancy risk).

#### 2.2.1 Diagnostic/treatment process and follow-up

The family received comprehensive counseling regarding the high suspicion of fetal TSC-associated CR, with formal recommendations for confirmatory genetic testing and detailed cerebral and cardiac imaging to stratify long-term prognostic outcomes. Nevertheless, the family explicitly declined all supplementary examinations and firmly requested termination of pregnancy. Induction was performed via intracardiac injection of 10% potassium chloride (6 mL) combined with amniocentesis of 100 mg rivanol (lactic acid ethacridine injection), followed by vaginal delivery of a stillborn female infant. The placenta was completely delivered, but the fetal membranes were incomplete, requiring manual curettage. Postpartum, 10IU intravenous oxytocin and 100μg intramuscular carbetocin were administered promote uterine contractility. The family declined fetal tissue sampling for genetic analysis. A follow-up ultrasound on postpartum day 3 identified intrauterine residual tissue with abnormal echoes, with a maximum diameter of 1.7 cm. The patient was discharged on postpartum day 4 with advice for re-examination 1 week later. The residual status after discharge was pending follow-up (Figure 2).

### 2.3 Case 3

A 28-year-old G1P0 female was admitted for induction at 24 + 3 weeks of gestation due to “a hyperechoic mass in the fetal left ventricle”. She had irregular menstrual cycles (30–60 days) and attended regular prenatal visits. In early pregnancy, she received Baotai Pills and dydrogesterone tablets for 1 month due to “gestational sac subchorionic hemorrhage”. NT was 1.2 mm, and NIPT showed low risk for common aneuploidies. Transabdominal ultrasounds at an external hospital (22 weeks) revealed “a hyperechoic mass in the fetal left ventricle (possible CR) and punctate hyperechoic foci in both ventricles”. Jining First People's Hospital's fetal echocardiogram confirmed “a hyperechoic nodule (4.6mm × 5.1mm × 3.3 mm) near the apex of the fetal left ventricular wall (possible CR)”. Amniocentesis was recommended but declined. She further consulted Shandong Maternal and Child Health Hospital, which still suggested prenatal diagnosis, but the patient and her family decided to terminate the pregnancy. No in-hospital ultrasound was performed. At admission, she reported no discomfort, with a total pregnancy weight gain of 10 kg. The pregnancy risk assessment was classified as green (indicating minimal pregnancy risk).

#### 2.3.1 Diagnostic/treatment process and follow-up

Although the family had initially declined prenatal amniocentesis, they firmly requested termination of pregnancy after thorough multidisciplinary counseling. Induction was conducted via intra-amniotic injection of 100 mg rivanol, followed by vaginal delivery of a stillborn male infant. The placenta was completely delivered, but the fetal membranes were incomplete, requiring manual curettage. Postpartum, the patient received Guizhi Fuling Capsules (4 capsules tid) to promote uterine involution and lower extremity compression therapy for prevention of deep vein thrombosis. A follow-up ultrasound performed on postpartum day 5 demonstrated intrauterine residual tissue with abnormal echoes, with a maximum diameter of 1.8 cm. A two-week clinical re-examination was scheduled for continuous maternal monitoring. Whole-exome genetic testing identified a pathogenic fetal TSC1 variant (NM_000368.4:c.1498C > T, p.Arg500*), which confirmed the diagnosis of TSC-related CR (Table 1).

**Table 1:** Clinical characteristics, genetic findings, and pregnancy outcomes of three cases with fetal cardiac rhabdomyoma.

Case No.	Maternal Age (years)	Gestational Age at Induction	Fetal Ultrasound Findings	Genetic Test Results	CR Etiology
1	25	27 + 2 weeks	Left ventricular apical hyperechoic nodule	TSC2 c.2286dupG; 46,XX,t(6;11)	TSC2 familial inheritance-related
2	30	38 + 1 weeks	Biventricular multiple hyperechoic nodules, the largest lesion in the right ventricle measures 1.3 × 1.0 × 1.2 cm	Refused by family	Undetermined
3	28	24 + 3 weeks	Left ventricular hyperechoic nodule (4.6 × 5.1 × 3.3 mm) (external ultrasound only; no in-hospital ultrasound)	TSC1 c.1498C > T (p.Arg500*)	TSC1 mutation-related

CR, cardiac rhabdomyoma; TSC, tuberous sclerosis complex; PGT-M, preimplantation genetic testing for monogenic disorders.

## 3 Discussion

### 3.1 Standardized diagnostic pathway and imaging evaluation of fetal CR

Fetal cardiac rhabdomyoma (CR) is the most prevalent benign cardiac tumor in the fetal population. On prenatal ultrasonography, this lesion typically presents as well-defined hyperechoic intracardiac nodules with sparse internal blood flow (5). All three cases enrolled in the present study exhibited these typical ultrasonic features, which were highly suggestive of CR. Nevertheless, prenatal diagnosis cannot rely solely on imaging manifestations, and rigorous differential diagnosis is essential to distinguish other fetal intracardiac space-occupying lesions and avoid clinical misdiagnosis. Cardiac fibroma usually manifests as a solitary, hypoechoic, well-circumscribed solid mass without invasive growth, whereas cardiac hemangioma is characterized by abundant intralesional vascularity. These imaging characteristics differ distinctly from the hypovascular nature of CR, enabling effective differential identification (6).

Transabdominal ultrasound remains the primary screening modality for fetal CR. Clinicians should systematically evaluate lesion size, boundary integrity, echo homogeneity, and blood flow distribution to acquire complete and standardized imaging data. For fetuses with highly suspected CR on routine ultrasound, targeted fetal echocardiography is recommended to comprehensively assess overall cardiac structure, biventricular systolic function, atrioventricular connection, and ventricular outflow tract patency. This standardized diagnostic workflow improves diagnostic accuracy and provides robust imaging evidence for subsequent stratified clinical management, prognostic evaluation, and pregnancy decision-making.

In accordance with the 2022 Tuberous Sclerosis Complex (TSC) perinatal guidelines (7), all suspected CR cases require centralized image review by experienced fetal medicine specialists. This procedure unifies tumor measurement criteria and morphological description standards to maximize diagnostic precision. Notably, maternal chromosomal structural abnormalities do not exclude a TSC-related pathogenesis, which renders genetic testing indispensable for etiological diagnosis (2). In Case 1, the mother harbored a balanced chromosomal translocation, while the fetal CR was ultimately confirmed to be caused by a paternally inherited TSC2 mutation. This clinical observation further verifies that pathogenic variations in TSC genes, rather than chromosomal anomalies, represent the predominant etiology of fetal CR.

### 3.2 Genetic stratification, clinical decision-making, and expectant management

Genetic testing lays the foundation for identifying fetal CR etiology, distinguishing sporadic cases from familial ones and evaluating recurrence risks, and also provides key evidence for prenatal genetic counseling and long-term reproductive guidance (8). Obvious genetic heterogeneity was observed among enrolled cases, and distinct inheritance patterns, recurrence risks and long-term prognoses existed across different mutation subtypes. Case 3 was diagnosed with a *de novo* TSC1 mutation, belonging to a sporadic case with a low recurrence risk of approximately 1% and relatively favorable prognosis (9). By contrast, Case 1 carried a heterozygous TSC2 mutation inherited from an asymptomatic paternal carrier. Following autosomal dominant inheritance, the risk of recurrence in subsequent pregnancies reaches 50%.

Cumulative clinical cohort studies have demonstrated definite genotype-phenotype correlations in TSC (3). Compared with TSC1 mutations, TSC2 mutations lead to more severe multisystem damage and higher lifelong risks of intractable epilepsy, intellectual disability and renal angiomyolipoma (10). Mechanistically, the complex formed by TSC1 and TSC2 proteins negatively regulates the mTOR signaling pathway and maintains normal cell proliferation and differentiation (11). Functional defects of TSC2 trigger more excessive activation of the mTOR pathway, thereby inducing extensive hamartoma proliferation and more severe systemic manifestations (12, 13). Whole-exome sequencing adopted in this study boasts higher sensitivity and wider detection range for novel pathogenic mutations than conventional Sanger sequencing. Consistent with updated TSC diagnostic guidelines (7), this approach enables accurate etiological confirmation and genotypic classification, supporting individualized prenatal risk stratification.

### 3.3 Stratified clinical decision-making and individualized induction strategies

Fetal CR is a benign hamartoma with extremely low risks of malignant transformation, invasive growth and distant metastasis. The tumor has distinctive spontaneous regression properties. Most isolated small lesions stay stable or shrink gradually during gestation and resolve spontaneously in the neonatal or infant period (14). Only multiple lesions or TSC-associated tumors may progress continuously, compressing cardiac structures and impairing cardiac function. Based on domestic and international fetal tumor management guidelines (15), standardized stratified treatment principles are applied for fetal CR management. Isolated small lesions with intact cardiac function, unobstructed outflow tracts and no multisystem involvement are managed with regular expectant surveillance without pharmaceutical or surgical intervention. For progressively enlarging lesions accompanied by cardiac compression risks, *in utero* targeted therapy with maternal oral mTOR inhibitors such as rapamycin can be cautiously administered after multidisciplinary assessment to restrain tumor growth (16). Pregnancy termination can be considered after thorough consultation for genetically confirmed TSC cases complicated with multiple cardiac lesions, severe cardiac dysfunction and extremely poor neurological prognosis.

Multidisciplinary team (MDT) consultation is mandatory for high-risk cases with multiple lesions, confirmed TSC involvement and potential cardiac or multisystem impairment. The team consisting of obstetricians, fetal cardiologists, clinical geneticists and neonatologists comprehensively weighs the advantages and disadvantages of expectant management, *in utero* intervention and pregnancy termination. Multidimensional assessment avoids one-sided judgment from a single discipline and standardizes clinical decisions (17). All termination decisions in this study were made on the basis of full MDT evaluation. Parents received complete counseling covering disease characteristics, genetic risks, long-term prognosis and alternative therapeutic options, and made autonomous choices after full informed consent to guarantee scientific and ethical clinical practice.

Induction regimens were formulated individually according to gestational age, fetal and maternal conditions. Intra-amniotic ethacridine lactate injection was adopted for pregnancies ranging from 24 to 28 weeks. This mature regimen applied in second-trimester termination delivers high success rates, stable uterine contraction and satisfactory safety outcomes (18). For the near-term case at 38 + 1 weeks complicated with multiple CR lesions and highly suspected TSC and poor neurological prognosis, combined intracardiac potassium chloride injection and ethacridine lactate administration was conducted to avoid unintended live birth, complying with safety criteria for terminating high-risk fetal malformations in late pregnancy (19). All procedures strictly complied with local ethical norms and national clinical guidelines; termination was performed solely for fetuses with severe genetic disorders and extremely poor prognosis, without any nonmedical indication. Ethically, late pregnancy termination is only performed for fetuses suffering from severe genetic disorders and irreversible disabling lesions with extremely poor prognosis after sufficient MDT discussion and written informed consent. No termination was implemented for non-medical reasons in this research.

### 3.4 Postpartum management and long-term reproductive guidance

Postpartum care for terminated CR-related pregnancies focuses on maternal physical recovery and long-term genetic follow-up. Clinicians monitor uterine involution regularly and conduct pelvic ultrasound examinations to detect intrauterine residues and hemorrhage. Personalized conservative medication or clinical intervention is carried out based on imaging results to ensure maternal rehabilitation.

Long-term genetic follow-up and reproductive counseling play vital roles in cutting familial recurrence risks and improving subsequent pregnancy outcomes (20). Carrier screening and multisystem examination are recommended for asymptomatic parents of TSC-affected families to detect subclinical lesions in skin, brain, kidney and heart, so that early intervention can be implemented to prevent long-term complications (21). Hierarchical reproductive guidance is formulated in line with mutation types. Preimplantation genetic testing based on next-generation sequencing is strongly recommended for families with hereditary TSC2 mutations to block vertical transmission of pathogenic genes (22). Excessive medical intervention is unnecessary for sporadic *de novo* TSC1 mutations with low recurrence risk, while enhanced pre-pregnancy counseling and routine prenatal monitoring are sufficient. Standardized pre-pregnancy evaluation and serial fetal echocardiography help identify recurrent cardiac tumors and structural abnormalities at an early stage in subsequent pregnancies (23).

### 3.5 Study limitations

This study presents several methodological and clinical limitations reflecting prevalent difficulties in current prenatal CR management.

All enrolled cases underwent pregnancy termination, which may introduce a slight selection bias. Distinct gaps exist between real clinical practice and standardized guideline-based treatment. After learning about TSC pathogenic mutations and irreversible adverse outcomes including neurological damage and multisystem hamartoma, most families opt for pregnancy termination after taking lifelong fetal health conditions, long-term rehabilitation costs and family care burdens into consideration. Meanwhile, families often refuse further imaging reexamination, genetic verification and *in utero* treatment. Such common clinical phenomena inevitably impair clinical data integrity and serve as a major subjective constraint in prenatal counseling and high-risk fetal disease research.

Moreover, this is a single-center retrospective study with a small sample size, which restricts the extrapolation of research conclusions. Long-term data concerning maternal reproductive outcomes and fetal postnatal prognosis are also absent. Further multi-center prospective studies with unified imaging and genetic testing standards are required to optimize standardized diagnostic and therapeutic protocols as well as long-term genetic follow-up systems for fetal CR. Optimized genetic counseling modes are also needed to enhance parental disease awareness and raise the completion rate of standardized prenatal examinations.

## 4 Conclusion

This study summarized the prenatal imaging manifestations, genetic features, and individualized perinatal management of fetal cardiac rhabdomyoma (CR). Although fetal CR is a benign cardiac hamartoma with spontaneous regression potential, TSC-associated multiple lesions indicate adverse long-term prognosis. Significant genetic heterogeneity exists between CR cases with TSC1 and TSC2 mutations, and TSC2 variants are correlated with more severe multisystem impairment. Standardized prenatal imaging, precise genetic stratification, and MDT assessment contribute to standardized clinical management and accurate risk evaluation for fetal CR.

## Data Availability

The original contributions presented in the study are included in the article/Supplementary Material, further inquiries can be directed to the corresponding author.
